# A qualitative study of independent fast food vendors near secondary schools in disadvantaged Scottish neighbourhoods

**DOI:** 10.1186/1471-2458-14-793

**Published:** 2014-08-04

**Authors:** Michelle Estrade, Smita Dick, Fiona Crawford, Ruth Jepson, Anne Ellaway, Geraldine McNeill

**Affiliations:** University of Aberdeen, Aberdeen, Scotland; Glasgow Centre for Population Health, Glasgow, Scotland; Scottish Collaboration for Public Health Research & Policy, Edinburgh, Scotland; MRC/CSO SPHSU, University of Glasgow, Glasgow, Scotland

**Keywords:** Fast food, Diet, Nutrition, Food policy, Adolescence, School meals, Neighbourhood, Food vendors

## Abstract

**Background:**

Preventing and reducing childhood and adolescent obesity is a growing priority in many countries. Recent UK data suggest that children in more deprived areas have higher rates of obesity and poorer diet quality than those in less deprived areas. As adolescents spend a large proportion of time in school, interventions to improve the food environment in and around schools are being considered. Nutrient standards for school meals are mandatory in the UK, but many secondary pupils purchase foods outside schools at break or lunchtime that may not meet these standards.

**Methods:**

Qualitative interviews were conducted with fast food shop managers to explore barriers to offering healthier menu options. Recruitment targeted independently-owned shops near secondary schools (pupils aged c.12-17) in low-income areas of three Scottish cities. Ten interviews were completed, recorded, and transcribed for analysis. An inductive qualitative approach was used to analyse the data in NVivo 10.

**Results:**

Five themes emerged from the data: pride in what is sold; individual autonomy and responsibility; customer demand; profit margin; and neighbourhood context. Interviewees consistently expressed pride in the foods they sold, most of which were homemade. They felt that healthy eating and general wellbeing are the responsibility of the individual and that offering what customers *want* to eat, not necessarily what they *should* eat, was the only way to stay in business. Most vendors felt they were struggling to maintain a profit, and that many aspects of the low-income neighbourhood context would make change difficult or impossible.

**Conclusions:**

Independent food shops in low-income areas face barriers to offering healthy food choices, and interventions and policies that target the food environment around schools should take the neighbourhood context into consideration.

## Background

Adolescence has been identified as a critical period in the formation of dietary habits and the development of long-term patterns in body weight [1, 2]. Obesity in childhood is a particular concern because it is linked to a range of poorer health outcomes tracking through to adulthood [3]. Data pooled from a nine-country study in Europe revealed that socioeconomic status (SES) is related to adolescent diet quality, and the association becomes stronger in northern European countries [4]. The study also found that nutrition knowledge among European adolescents was “modest” at best and concluded that interventions targeting lower-income groups are needed [5].

Family environment plays an important role in shaping diet and lifestyle, but many children spend a considerable proportion of time away from home in, and beyond, the school environment. It is in such settings that the ability to make autonomous choices is developed and exercised. Numerous countries have school meal nutrient standards that are statutory and well-defined [6] but may not reach children who bring food from home or purchase lunch outside of school. In Scotland, 63% of secondary school students report purchasing food and beverages outside of school at lunchtime at least occasionally [7]. An observational study in Glasgow noted that food outlets catered specifically to pupils by offering them special lunchtime meal deals and promotions [8]. Subsequent analysis of the foods offered revealed that many exceeded recommended levels of energy, fat, and saturated fat, and salt. Further afield, a Brazilian study found that shops with more processed foods were closer to schools than those selling minimally-processed foods [9], and data from the Health Behaviour of School Children (HBSC) surveys in Canada showed a significant association between food retailer density within walking distance of schools and the probability of pupils eating lunch from those nearby retailers [10].

Policy efforts in a number of countries have been aimed at addressing obesity in a public health context, for example though mandatory calorie labelling on menus, restrictions on advertising of unhealthy foods to children, taxes on sugar-sweetened beverages or high-fat foods, limitations on serving sizes, and a ban on trans fats [11]. In May 2013 the Scottish Government issued a draft framework, entitled Supporting Healthy Choices [SHC] [12], encouraging food vendors in Scotland to offer healthier options, particularly to children and young people. The document details a wide range of recommended changes in cooking practices, promotional activities, portion sizes, as well as types and portion sizes of foods offered. If implemented, it is envisaged that such changes could have a potentially beneficial impact on the nutritional quality of food bought by pupils who eat outside of school at lunchtime. While a similar effort, known as the Responsibility Deal [13], has been tried in England, it has consisted almost exclusively of larger franchises signing on. Little is known about the way in which smaller, independent food businesses view these kinds of public health nutrition guidelines, nor the reasons they might have for not signing on to such initiatives.

This aim of this study was to identify the challenges that owners of small (independent) food businesses might face in making changes to improve the nutritional quality of their menu options. Given that interventions targeting lower-income groups are needed [5], we focused our data collection in less-affluent areas around Scotland.

## Methods

The research team ensured that this study was conducted and reported in accordance with the RATS guidelines for qualitative research [14].

### Setting

Lower-income areas around two secondary schools (pupils aged c.12-17) were selected in each of three Scottish cities: Aberdeen, Edinburgh, and Glasgow. Schools were selected on the proportion of pupils entitled to free school meals [15], which is based on household income. Schools were classified by this measure of household poverty rather than using area-level deprivation index, as a school’s location may not necessarily coincide with the socio-demographic profile of its pupils, given that secondary school pupils come from a wider catchment area than the area immediately surrounding the school. Geographic Information System [GIS] maps, built on those created for a previous study geocoding food retailers’ proximity to schools [16], were used to identify takeaway shops within easy walking distance (~1 km via road network, around a 10 minute walk) of schools where the proportion of pupils entitled to free school meals was above the national average. On-the-ground observations were carried out to confirm that locales meeting these inclusion criteria were also open for business during school lunch hours.

### Sampling and recruitment

A purposive sample of independent establishments selling foods prepared on site for take-away consumption during the school lunch period were selected. Chain restaurants and shops (those with multiple locales, in which site managers did not have executive decision-making power) were excluded as managers at those sites were expected to have limited control over menus and pricing: this was confirmed by speaking with on-site managers of two popular chains while recruiting. A total of fourteen shops were excluded for this reason. Recruitment materials were delivered to every shop meeting the inclusion criteria (total of 35) within the defined recruitment areas by a member of the research team (ME).

In total, 35 invitations were distributed. Recruitment packs contained a user-friendly expression of interest form, along with a pre-stamped, addressed envelope. Only one response was received, which was from a vendor who did not want to participate. In-person follow-up visits to each shop, typically three or four visits per shop, were then made in order to obtain interviews. The most common reason given by food vendors for declining participation was a lack of time or extra personnel to cover the duties of the manager/owner while s/he was being interviewed. Though we initially planned for 15–20 interviews, data saturation was reached quickly, such that no new codes were derived from the final two interviews, and further efforts to recruit were halted after 10 interviews.

### Data collection

Ethical approval for the study was obtained from the College of Life Sciences and Medicine Ethics Review Board at the University of Aberdeen prior to commencing recruitment. Informed consent was obtained in writing from each vendor prior to being interviewed. All interviews took place on the premises of the takeaway shops and lasted approximately ½ hour. Those who completed the interview received a £15 grocery gift card in acknowledgement of their time sacrifice. Interviews followed a semi-structured topic guide to explore food vendor experiences and views on making healthy changes to menus. Recommendations within the SHC framework formed the basis for many of the questions. Examples of the topics covered included: factors affecting decisions about what to offer on the menu and what price to ask; the meaning of “healthy eating” and its role in a takeaway environment; the potential role of NHS Health Scotland’s Healthy Living Award [17]; and the feasibility of specific recommendations within the SHC framework. During the interviews, further probing questions were asked within each discussion topic to elicit deeper responses about the habits and preferences of schoolchildren as customers.

### Analysis

Interviews were audio-recorded and transcribed verbatim, and transcripts were loaded in to NVivo 10 [18] for coding and analysis. Given the emergent nature of the data and the importance of letting participant voices be heard, a data-driven inductive thematic analysis method was used, allowing themes to develop directly from the data in the absence of any pre-existing model or framework. A total of 39 codes were identified and then grouped together by conceptual similarity. A theme was developed for each group of codes, chosen as a word of phrase that would best describe and encompass the essence of the codes in that group. Constant comparison was used to ensure representation of all perspectives, and negative cases as well as unanticipated themes were sought [19, 20]. Interviews, transcription, and coding were all undertaken by a single researcher (ME), ensuring the highest possible level of acquaintance with the data. A second researcher (SD) independently coded a random selection of interviews for comparison, in order to confirm coding validity. Initial coding agreement was over 87%, and the remaining codes were settled upon with input by a third researcher (RJ).

Data saturation was reached by the 8^th^ interview. Ninety-two per cent of the codes had been established after seven interviews, and no new codes emerged from the final two interviews.

## Results

Nine of the interviews were conducted with managers or owners of the participating businesses, and one was conducted with a senior employee familiar with the workings of the business. Although more of the invitees were male, a greater number of female interviews were obtained. Forty per cent of all female invitees agreed to an interview, while only twenty per cent of male invitees did so (Table 1).

**Table 1:** **Recruitment statistics**

Establishment type	Invited	Interviews attained
Café/sandwich/snack shop	16	5
Kebab	7	2
Fish & Chipper	6	2
Chinese	3	0
Indian	2	1
Mobile van	1	0
**Total**	**35**	**10**
**Invitee Gender**		
Male	20	4
Female	15	6

### Themes

Five overarching themes emerged throughout the interviews. These were: pride in what is sold; the concept of individual responsibility for one's health; customer demand; profit margins; and the unique underpinnings of the neighbourhood context. Words in brackets have been added to some quoted material in order to clarify meaning. For differentiation, a unique code assigned to the individual vendor appears before each quote.

#### Pride in what is sold

The independent food vendors interviewed in this study consistently took pride in the food they sold, most of which they made themselves. They used words like *traditional*, *proper*, and *fresh* to describe their menus, and viewed themselves proudly as an integral part of the fabric and identity of the neighbourhoods in which they did business. Belief that they were preparing food the “proper” way was one important factor affecting their willingness to make changes to cooking methods.

[v1] Beef dripping is what we cook in and it’s… I mean how can we say we’re traditional if we’re cooking in palm oil? Palm oil is a new thing, or rapeseed oil, it’s a new one, and that’s not the taste. It does affect the taste.

While vendors recognised that diet quality and healthy eating were public health concerns, they tended to believe that they were selling healthier foods than their competitors, and that other shops in the neighbourhood were the ones in need of making changes.

[v1] …they’ve proven like a fish & chips is healthier than, well it’s got less calories and stuff in it than your kebabs or your pizzas.

[v2] …well actually this kebab shop is more healthy than chippie shops. Fried things, you know they cook it in the fat, full fat oil and everything, and this is like, unhealthy, you know? We cook mostly pizzas and kebabs and grill, and it's very healthy, you know… [fat] going down with pure meat left, and salad. It’s very healthy.

#### Personal autonomy and responsibility

Belief in individual and personal responsibility was a broad and cross-cutting theme, coming out in every interview across a range of general questions about the meaning of healthy eating, as well as specific questions about the SHC framework.

Most vendors felt they already provided opportunities for healthy eating, but that customers who purchase the less healthy options would not be persuaded to change their food preferences, especially school children. The vendors consistently saw themselves as external to customers’ preferences and felt they had very little influence in a customer’s decision-making process.

[v3] …if somebody wants to eat fried chicken, you can’t convince him, no: go for grilled…

Several were keenly aware of the negative press directed at takeaways in relation to obesity and conveyed a sense of antipathy to that notion.

[v1] Obesity starts from… you've got to get into values. I mean, I’m an overweight woman. I know why I’m overweight. It’s because I eat too much. It’s not rocket science. Everybody knows that. You just can't blame a company or whatever, because it's what I choose to eat.

[v5] I usually like to give enough food for the customer, so it's up to them to make up their own mind about how much they eat… I mean when you reach 18 years old, even before that, you're responsible for what you put in your stomach… If you want to eat well, you will eat well.

[v8] Is it not [enough] just reducing their portion sizes when they’re home?

#### Customer demand

Vendors were keenly aware of customer preferences and felt they needed to offer what customers perceived as “good food” and in order to keep their business. They conveyed a strong belief that simply offering healthier options wouldn’t create a shift in customer demand, viewing themselves as relatively powerless to change what people want.

[v4] No. I don’t think we have the influence [on what customers eat]. I think people have got their favourite kind of food…and if you’re a good business, then yes, your reputation will bring people back. So, I think that would be the influence. Good food.

[v3] …the people who are health conscious, they only think about that. The people who are not, they don't bother you even with 100 different options, they're gonna pick the Coke out of it. You know, so this is not actually the question, what is available, what is not available. …if somebody wants something, they come here to buy that thing. They don't come here to [say] “okay, don't give me this, you can give me that”.

Several managers with school-aged children or grandchildren expressed their frustration at the preferences and purchasing habits of school children who entered their shops at lunchtime. Many also felt that offering healthier options to school children would not be enough to produce any real change in behaviour.

[v6] School kids eat chips. And baguettes. That's it. They dinnae [don’t] want anything else but chips and curry sauce or chips and cheese, and a baguette.

*[interviewer] What about offering a healthy meal deal or discount?*

[v6] I would like to do that with the kids, because we do salad boxes. I'd like to do that. But it's just, they cannae [can’t] see past chips. I'd like to do an offer on the salad boxes, but it's just they want chips.

*[interviewer] Do you do special offers for the kids?*

[v7] Yeah we do, but it’s a healthy option, it’s a sugar-free juice, baked crisps, and a tuna roll.

*[interviewer] Do they go for that?*

[v7] No! …we’re lucky if we sell one a month!

A sense of niche was an important part of why vendors perceived that their customers would not be receptive to more healthy options from their outlet.

[v4] …certainly if there was anything like a deal with a healthier option, people prefer to go to a sandwich shop for sandwiches and come to a chipper for chipper food… As I say, people are coming to the chip shop and they’re coming because they want… they’re not coming because they want fruit.

One vendor described the problem as one that is influenced by more upstream factors.

*[interviewer] If you have a meal deal and in place of crisps you give fruit, and in place of fizzy drink you offer water, would people go for that?*

[v10] No! Ha! No! I think you have to get the kids when they’re young to build it up. And I think, because there’s quite a lot of poverty, they just go for the cheapest and the most filling. And that’s what the problem is… it’s instilled in them…

Some also felt that offering too many options could put customers off.

[v8] I think the customers might start getting confused because there’s just too much choice. I think that can be off-putting as well. I think we’ve kinda discovered that, even after all these years, that too much choice people don’t like. They like a limited [choice] so that it’s easy for them to pick.

#### Being profitable

Cost and profitability were major concerns among food vendors, especially in regards to introducing healthier options. Price was seen as playing a central role in customers’ decisions about which menu items to choose, and most vendors cited it as the key factor in their own decisions about which products to purchase for their shop and from which suppliers. They described a constant struggle to cope with the economic pressure of rising food costs and tightening profit margins. Many vendors also cited difficulty in finding the right balance between increasing menu prices and keeping their customers.

[v4] Prices have gone up. Even like potatoes. I mean at this time last year… we're getting three times the price of potatoes to what we were last year because of all the floods in England and that, so, and the fish is scarce, so that's gone up considerably. So that's your two main things you use in this kind of business, so yeah, it's kinda costly… And I think just with the recession and that people are kind like choosing something a bit cheaper because they can't really afford these expensive meals.

Healthier options were viewed as unfeasible in many cases, because vendors perceived that they wouldn’t be able to charge enough to make a profit.

*[interviewer] Do you think there’s any way they could be enticed to buy [healthier options]?*

[v10] I don’t know, you’d probably have to give them away for nothing…

[v6] I don't allow my [own] kids to have fizzy juice [carbonated soft drinks], so I dinnae [don’t] really like having it. I'd like to buy fresh juice, but it's the prices… it's so expensive, and you wouldn't make any money. With the price you would have to charge, they would nae [not] want to buy it. So you've got to think of that as well… A case of Coke for seven pound, you would never buy juice for that price; no way… If the government wants you to buy all this fresh stuff, they should bring down the price.

Because vendors were struggling to turn a profit in order to stay in business, a culture of competition was also evident in many of the neighbourhoods, including rivalry for pupils’ lunchtime business.

[v6] They just want chips… they go about looking for the deals; they'll have a look and then go along have a look at their deals and then come back and they'll order… they like the value for money. The competition here is unbelievable.

#### Neighbourhood context

In all three cities, interviews were conducted in neighbourhoods that were comparatively less well-off than many others in their city. Though shops were chosen for their proximity to schools in which a higher than national average proportion of children were receiving free school meals, the Scottish Index of Multiple Deprivation score confirmed the status of each neighbourhood as more deprived. Food vendors sensed this, and though the economy in general was implicated as a negative factor, the unique and enduring characteristics of each neighbourhood were viewed as an added burden in the struggle to make a profit.

[v2] we are still selling the same things, you know, same prices… if you put it too high… oh! Customers say it's too expensive. It's too expensive….in *this* area. In some rich areas people don't complain…

[v3] it's very hard. A takeaway business is very hard. To get a customer and keep it, it's very hard… There's already so much struggling in this, particularly in this area, you have to survive very hard…the business is struggling… There are like four takeaways just on this street which are empty.

[v1] the prices have gone up so much where our profit margin… we're just barely making a profit just now. If we were probably anywhere else in Aberdeen or in the city centre we'd be able to charge more, but in [this neighbourhood], most of our customers are on a limited budget. We just can't, we're just pared right down to the bone.

Most vendors were not keen to seek out publicity for healthy menu options. They did not believe that a Healthy Living Award [17] would have any positive impact on their business or customers’ perceptions, and that it might even send mixed messages.

[v1] I think that would be a bit of a joke… people would be laughing at that in a chip shop.

[v3] If you're sitting on Princes Street [centre of Edinburgh], posh people walking in, they see this [HLA] sticker in the window, yes, they pick like that. Yes, it does matter. Because their sort of perception, you know… they want to see those sort of things. But the area we live in… they don't care what you display.

However, one vendor commented that it could be a good way to attract couples or groups who want to eat together but have varying priorities and preferences when it comes to healthy eating.

### Regulatory levers

An alternative approach being implemented in various countries is use of regulatory levers involving health-related food taxes, although with the exception of sugar sweetened beverages [21] there is little evidence based on rigorous evaluation [22, 23]. During interviews in the current study, we discussed food taxes with the vendors. They tended to be strongly opposed to this approach because they felt that it would be an unfair burden on small business. In particular, because of the neighbourhood context and their hesitancy to ask higher prices of their customers, vendors believed that they would have to bear the cost of taxes targeting specific foods.

[v1] Supermarkets can absorb it. We can't. We can't absorb that kind of tax. It’s crazy. I can't see how that would influence obesity.

Vendors also felt that any increase in price might drive customers to purchase the more expensive foods elsewhere.

[v8] I don’t think that is gonna do anything [for obesity]. It’s just gonna affect our business. You know, because they’ll maybe not buy it from here, but they’ll get it cheaper at the supermarkets.

## Discussion

The aim of this study was to identify potential barriers that independent fast food vendors near lower-income secondary schools might face in offering healthier menu options. The food vendors in this study were proud of the foods they made and sold, and viewed their offerings as healthier than their competitors’. While they recognised that obesity is a major public health problem, vendors felt that both the cause and resolution are rooted in individual responsibility for one’s diet and lifestyle choices. They explained that, as with non-food businesses, responding to customer demand is a priority, and that customers in their area, especially school children, would not be persuaded to purchase healthier foods. Vendors in these relatively low-income neighbourhoods expressed concerns about offering menu items that wouldn’t sell well, because they were already struggling to maintain a profit and stay in business. When explaining why it would be unfeasible for them to offer healthier options or change their customers’ behaviours, they cited characteristics rooted in culture and the neighbourhood context.

The views expressed by food vendors in this study are similar to those from a London study that examined independent food vendors’ views on implementing better sustainability practices. The vendors felt unable to make changes to their business practices because profit margins were too tight to risk a drop in customer demand [24]. In our study, vendors’ views about individual responsibility for eating habits, body weight, and health in general were also consistent with current cultural norms. When members of a large restaurant association were surveyed, health and nutrition issues were reported by less than 1% of restaurant owners and managers as topics of concern [25], and a recent survey of British adults found that nearly two thirds believed that being overweight was an individual’s own fault, attributable to a lack of willpower [26].

The vendors we interviewed felt they occupied a special niche in their neighbourhoods with the type of foods they offered and were reluctant to change those offerings. They were also reluctant to add healthier options (as an alternative to *replacing* current menu items) because they felt that too much choice would put customers off. Current literature in this area indeed supports the idea that too much choice can have negative psychological effects for customers [27].

In their study on features of convenience stores surrounding schools in Minnesota, USA, Gebauer et al. [28] identified the following as barriers to stocking healthy foods: challenge to switch over stock, lack of infrastructure, and fear of losing business. All of these have been expressed as concerns in the current study by the food vendors interviewed. In addition, the vendors we interviewed cited lack of technical knowledge that would be needed to implement the SHC guidelines, for example in order to offer calorie information to customers. Several UK cities have had success offering this type of training to fast food vendors as part of local healthier food awards schemes [29, 30]. However, because vendors in our study mentioned that healthy food awards were not important to their customers, alternate incentives may be needed to reach vendors in deprived areas. As an example, a scheme implemented in Northern Ireland offered a £200 grant to businesses who took part in the Healthier Takeaways Project [31]. The program was successful in helping fast food vendors to make small, sustainable changes, like replacing 17-hole salt shakers with 5-hole shakers in order to reduce levels of added salt.

Although the idea of regulation was opposed by most vendors in our study, it could be a useful tool to “level the playing field” and ease the pressure on vendors to compete. One current approach being discussed is the possibility of policies to limit targeting and promotional strategies that vendors utilise to attract school pupils as lunch-time customers [32].

In addition to targeting food vendors, interventions that target consumer behaviour may be necessary to influence customer demand to which vendors respond. Further research is needed to identify the factors that most influence the food choices of pupils in deprived areas. It is worth noting, however, that school children make up only a fraction of the clientele for the food vendors we interviewed. Vendors must take the preferences of the wider community that they serve into account, and therefore targeting pupil behaviour may not be enough.

Examining the situation through an economic lens can also help explain the positions taken by food vendors in this study. Chell and Pittaway [33] explored the ways in which restaurant and café entrepreneurs talked about their businesses and found large differences in the actions taken by expanding businesses compared to declining businesses. Expanding businesses described measures that characterised proactive behaviours, such as making menu changes. Declining businesses described measures indicative of reactive behaviour, such as making changes based on an Environmental Health officer’s recommendations. Given that the businesses we interviewed described themselves as struggling to survive, it is logical to question whether or not these businesses can be expected to implement proactive changes without clear evidence of potential economic benefit.

Time has been cited in a number of studies as an important factor of influence on food choice [34], and it has been described by schoolchildren as a concern of higher importance than cost [35]. Therefore, we expected time to come out as a strong theme that vendors recognised as important in attracting customers, particularly at school lunch time, but it was only mentioned briefly in two out of the ten interviews. It became clear through our own observations, as well as from the comments of the food vendors, that the schoolchildren in these neighbourhoods are willing to spend their lunch time in longer queues to get the best deal on the foods they want. One possible explanation is that within the deprived neighbourhood context, time may become a less important aspect than price, since previous research in low-income communities has found that cost is a major barrier to healthy food choices [36]. When children from a deprived London neighbourhood were asked what would motivate them to make healthier choices at takeaways, they cited cheaper prices and a better choice of foods, including fruits and vegetables [37], and a study in Glasgow produced similar findings [32]. To inform the planning of targeted interventions, further in-depth follow-up among schoolchildren in disadvantaged neighbourhoods will be useful.

The hard-to-reach population represented in this study makes it unique. Because independent food vendors in low-income areas are a very difficult group to recruit, this is one of few studies that has involved them directly and brought their opinions and concerns to light. However, the results found here may not be generalizable to food vendors in other countries, higher-income areas, or to mainstream shops such as supermarkets and food outlet chains, which are also frequented by schoolchildren and may have different strategies, priorities, and concerns in regards to menus, pricing, and profit margins. Further, because the shop owners in this study (and others [38]) were willing to spend only a short period of time in the interviews, it is possible that data saturation was reached quickly due to feasibility limitations on depth. Our study was conducted in three Scottish cities, which may vary in their local culture, norms and values but what was striking about our findings was the extent to which the same issues were mentioned by vendors we interviewed across the three cities.

## Conclusions

Interventions and policies that target the food environment around schools should take the neighbourhood context into consideration. Independent food shops in disadvantaged areas may face significant barriers to offering healthy food choices, and implementing voluntary guidelines may inadvertently exclude shops in disadvantaged neighbourhoods from feasible participation, thereby potentially widening current inequalities. If voluntary frameworks are to be implemented in economically disadvantaged areas, independent food vendors may need substantial financial and technical assistance to ensure that their customers don’t miss out on potential benefits.
